# Dexamethasone and Doxycycline Doped Nanoparticles Increase the Differentiation Potential of Human Bone Marrow Stem Cells

**DOI:** 10.3390/pharmaceutics14091865

**Published:** 2022-09-04

**Authors:** Manuel Toledano-Osorio, Sergio López-García, Raquel Osorio, Manuel Toledano, David García-Bernal, Sonia Sánchez-Bautista, Francisco Javier Rodríguez-Lozano

**Affiliations:** 1 Faculty of Dentistry, University of Granada Colegio Máximo de Cartuja s/n, 18071 Granada, Spain; 2 Medicina Clínica y Salud Pública Programm, University of Granada, 18071 Granada, Spain; 3 Departament d’Estomatologia, Facultat de Medicina I Odontologia, Universitat de València, 46010 Valencia, Spain; 4 Hematopoietic Transplant and Cellular Therapy Unit, Faculty of Medicine and Odontology, IMIB-Arrixaca, University of Murcia, 30120 Murcia, Spain; 5 Department of Health Sciences, Catholic University San Antonio of Murcia, 30107 Murcia, Spain

**Keywords:** cell proliferation, dexamethasone, doxycycline, nanoparticles, osteogenic differentiation, stem cells, zinc

## Abstract

Non-resorbable polymeric nanoparticles (NPs) are proposed as an adjunctive treatment for bone regenerative strategies. The present in vitro investigation aimed to evaluate the effect of the different prototypes of bioactive NPs loaded with zinc (Zn-NPs), doxycycline (Dox-NPs) or dexamethasone (Dex-NPs) on the viability, morphology, migration, adhesion, osteoblastic differentiation, and mineralization potential of human bone marrow stem cells (hBMMSCs). Cell viability, proliferation, and differentiation were assessed using a resaruzin-based assay, cell cycle analysis, cell migration evaluation, cell cytoskeleton staining analysis, Alizarin Red S staining, and expression of the osteogenic-related genes by a real-time quantitative polymerase chain reaction (RT-qPCR). One-Way ANOVA and Tukey’s test were employed. The resazurin assay showed adequate cell viability considering all concentrations and types of NPs at 24, 48, and 72 h of culture. The cell cycle analysis revealed a regular cell cycle profile at 0.1, 1, and 10 µg/mL, whereas 100 µg/mL produced an arrest of cells in the S phase. Cells cultured with 0.1 and 1 µg/mL NP concentrations showed a similar migration capacity to the untreated group. After 21 days, mineralization was increased by all the NPs prototypes. Dox-NPs and Dex-NPs produced a generalized up-regulation of the osteogenic-related genes. Dex-NPs and Dox-NPs exhibited excellent osteogenic potential and promoted hBMMSC differentiation. Future investigations, both in vitro and in vivo, are required to confirm the suitability of these NPs for their clinical application.

## 1. Introduction

Periodontitis and peri-implantitis are multifactorial diseases with an infectious and immunologic component. The main indicator of these diseases is the surrounding tissues’ chronic inflammation, which can also include the gradual resorption of alveolar bone. Both diseases generate a continuous and complex inflammatory response, mediated by the recruitment of various types of immune cells to the region and induce activation and polarization of monocytes toward pro-inflammatory macrophage phenotype (M1) [1,2], which subsequently will alter and impede the normal bone healing process [2]. 

The strategy for controlling these pathologies has been mainly the classical anti-infectious management through scaling and root planing, with the goal of removing the dental biofilm [3]. Periodontal surgery is occasionally required to regenerate these bone defects, which are challenging due to their unpredictable clinical results and can only address a small proportion of the defects [2,4]. Therefore, new strategies in bone tissue engineering able to create a natural bone healing environment is a promising approach for regenerating these hard tissues. Recent research about biomaterials and tissue engineering in this area has emerged with attempts to augment the natural healing capacity of alveolar bone [5]. A biomaterial able to produce an osteogenic effect capable of recruiting immature cells and stimulating them to differentiate into preosteoblasts and, ideally, with antibacterial and immunomodulatory properties is desired.

Contamination and infection are the events that usually hamper the success of regenerative bone treatments. Trying to overcome this situation, the use of controlled liberation of local antimicrobials has been previously described [6]. It is necessary to focus on developing a biomaterial with the controlled release of therapeutical substances [7]. Metal ions, such as zinc, have been employed as antimicrobial agents against periodontal pathogens [8]. Regarding antibiotics, tetracyclines have been the most recommended for this purpose since they are broad spectrum. They have efficient bactericidal activity against frequent periodontal pathogens, a low bacterial resistance rate, relative body temperature stability, tissue compatibility, and a low resorption rate [8,9]. 

However, our efforts should not only focus on the infectious side of the disease; the immunological approach should also be addressed. Understanding the immunopathological processes of periodontal and peri-implant diseases would help to include the immunomodulatory approach in the therapeutical arsenal against these diseases [10,11]. For this purpose, the use of dexamethasone has been proposed. This glucocorticoid is not only an immunomodulator and has anti-inflammatory properties but is also an osteogenic drug used for cell culture experiments to induce proliferation, maturation, and extracellular matrix mineralization of osteoblasts [12].

In order for biomaterials to achieve a controlled release of drugs, nanotechnology has gained relevance in medicine and dentistry, and different nanostructured materials have been researched for the treatment of periodontitis and peri-implantitis [13,14]. Non-resorbable polymeric nanoparticles (NPs) have been proposed [15]. These NPs exhibit carboxyl groups on their external surface, which may be functionalized with different ions or molecules [16,17,18], thus producing desired biological properties. Present polymeric NPs can effectively be loaded with zinc (Zn-NPs), doxycycline (Dox-NPs), or dexamethasone (Dex-NPs) [8]. Antimicrobial activity in subgingival biofilms, when grown on hydroxyapatite or onto titanium surfaces, has previously been demonstrated, which makes their use a potentially effective tool adjunctive to the classical antibacterial treatment [8,17].

It should be taken into account that osteogenesis is, therefore, crucial during bone regeneration and remodelling [19]. In this process, osteoblasts emerge from the differentiation of osteogenic progenitor cells and are responsible for the synthesis and mineralization of bone during initial bone formation and later bone remodelling [20,21]. Therefore, osteoblastogenesis, that is, the differentiation of osteogenic cells (human bone marrow mesenchymal stem cells (hBMMSC)) into osteoblasts, is essential for bone regeneration.

This work aimed to evaluate if zinc, doxycycline, and dexamethasone-doped polymeric nanoparticles exert effects on hBMMSC viability, proliferation and differentiation through the conduction of an exhaustive battery of assays.

## 2. Materials and Methods

### 2.1. Preparation of Experimental Nanoparticles

The experimental NPs were produced through a process of polymerization/precipitation, as previously described by Osorio et al. [16]. They were composed of 2-hydroxyethyl methacrylate (backbone monomer), ethylene glycol dimethacrylate (cross-linker) and methacrylic acid (functional monomer). The hydrodynamic size distributions and polydispersity index of the NPs measured by dynamic light scattering in distilled water were previously assessed. NPs have a mean particle size of approximately 250 nm, and the polydispersity index was 0.05 [22,23]. A detailed description of the NPs fabrication and characterization is at Medina-Castillo et al. [15]. Then, some of the NPs were functionalized to study the four different types of NPs: (1) Undoped NPs (NPs); (2) NPs loaded with zinc (Zn-NPs); (3) NPs loaded with doxycycline (Dox-NPs); and (4) NPs doped with dexamethasone (Dex-NPs). For the doping process of Zn, 30 mg of NPs were immersed for three days at room temperature and under continuous agitation (rotator Orbit 300445, JP Selecta, Barcelona, Spain) at 12 rpm, in an aqueous solution of ZnCl_2_ (containing zinc at 40 ppm, at pH 6.5). For doping NPs with doxycycline and dexamethasone, 30 mg of NPs were submerged in a 40 mg/mL aqueous solution of doxycycline hyclate or sodium dexamethasone (pH 7). NPs were maintained for 4 h under constant shaking (rotator Orbit 300445, JP Selecta, Barcelona, Spain) at 12 rpm. The NPs were then centrifuged (Centrofriger BLT, JP Selecta, Barcelona, Spain) at 6000 rpm for 30 min and the particles were separated from the supernatant and washed. The same centrifugation procedure was repeated twice, adding PBS solution for washing purposes. Finally, the NPs were separated from the supernatant and dried in an oven at 45 °C (Selecta, JP Selecta, Barcelona, Spain) until constant weight. As previously shown, the size of NPs did not change after loading, and no agglomeration was produced [23]. Loading efficacy and drug release kinetics up to 28 d of zinc and doxycycline were previously measured by inductively coupled plasma optical emission spectrometry and high-performance liquid chromatography, respectively. Relevant data may be found elsewhere [16,18].

### 2.2. Cell Harvest and Isolation

Adult hMSCs were isolated from bone marrow using a previously reported protocol [24]. All experimental protocols were in accordance with the Declaration of Helsinki and were approved by the Ethics Committee of the Virgen de la Arrixaca University Hospital (ID: 101212/1/AEMPS). The informed written consent forms were obtained from all the patients. For isolation, the aspirated material was placed into transfer bags containing heparin. The mononuclear cell fraction was obtained using Ficoll density gradient media and a cell-washing closed automated SEPAX™ System (Biosafe, Eysines, Switzerland). Then, the cell suspensions were cultured and expanded in Alpha Modified Eagle’s Minimum essential medium (α-MEM) (UFC Biotech, KSA) containing 10% fetal bovine serum (FBS), 2 mM L-glutamine, and a mix of 100 units/mL penicillin with 100 μg/mL streptomycin (P/S) at 37 °C and 5% CO_2_. The hBMMSCs from the third to sixth passages were used in the experiments.

### 2.3. Analysis of hBMMSC Viability Exposed to NPs

The viability of the hBMMSCs exposed to nanoparticles (NPs) was quantitatively assessed with a resaruzin-based assay according to the manufacturer’s recommended protocol (Alamar blue, ThermoFisher, Waltham, MA, USA). The hBMMSCs were seeded in 96-well plates at a density of 8 × 10^3^ cells/well in α-MEM containing 10% FBS and 1% P/S, and incubated for 48 h to attain cell confluence. After that, the cells were exposed to NPs (0.1, 1, 10, and 100 µg/mL), Zn-NPs (0.1, 1, 10, and 100 µg/mL), Dox-NPs (0.1, 1, 10, and 100 µg/mL) and Dex-NPs (0.1, 1, 10, and 100 µg/mL) based on the previous viability assessments carried out in the pilot phase of the study (data not shown). Cells without NPs served as the control group. Cell viability was analyzed at 24, 48, and 72 h of culture with NPs. Fluorescence intensity was analyzed using spectrophotometry at 570 nm. Three independent experiments were performed in which three samples per group were tested.

### 2.4. Cell Cycle Analysis

For cell cycle analysis, the DNA content was submitted to flow cytometry to distinguish which cells were in each cell cycle phase. The cells were cultured in 25 cm^2^ culture flasks at a density of 1 × 10^5^ cells per well in the presence of the different NPs prepared at several concentrations (0.1, 1, 10, and 100 µg/mL) for 72 h. After being centrifuged at 400× *g* for 4 min, cells were collected and fixed with 70% ethanol overnight at 4 °C and incubated with 40 μg/mL of propidium iodide and 200 μg/mL RNase for DNA content analysis. Finally, stained cells were immediately analyzed with a FACSCanto II Flow Cytometer (Becton Dickinson, San Jose, CA, USA) applying an excitation wavelength of 488 nm and an emission wavelength of 617 nm. The percentage of cells in G_0_/G_1_, S, and G_2_/M phases were analyzed using CellQuest and Modfit LT programs (Becton Dickinson). Tests for each experimental condition were performed in triplicate, and then they were analyzed in three independent experiments.

### 2.5. Cell Migration Evaluation (Scratch Wound Assay)

A horizontal scratch wound healing assay was carried out to assess the migration ability of hBMMSCs in response to the several NPs. The hBMMSCs were seeded at a concentration of 2 × 10^5^ cells in a 12-well plate. Thereafter, a 200 μL pipette tip was used to scratch through the confluent layer of cells and they were exposed to the diverse NPs and concentrations (0.1, 1, 10, and 100 µg/mL): no NPs were added to the control group. Wound closure was analyzed at three time intervals: 0–24 h (first time interval), 24–48 h (second time interval), and 48–72 h (third time interval). Phase-contrast microscopy (Olympus, Japan) was used to take images of the wound area after scratching. Twelve acquired images per group were used for analysis by ImageJ v. 1.53e software (National Institutes of Health, Bethesda, MD, USA). To account for the width variations among the scratch wounds, migration rates were presented as percentage areas of relative wound closure (RWC) and calculated as follows: RWC (%) = (wound closure area (pixels)/total number of pixels) × 100.

### 2.6. Cell Morphology and Spreading

Cell morphology and spreading were assessed by fluorescent-phalloidin labeling. A density of 3 × 10^4^ cells was seeded in a 24-well plate, allowed to adhere, spread, and cultured in untreated medium (control) or NPs for 72 h at 37 °C. Then, 1 mL of 4 % paraformaldehyde (PFA) solution was added to each sample for 15 min at room temperature to fix the hBMMSCs on the surface. They were then permeabilized with 1 mL of Triton X-100 (Solarbio, Beijing, China). The cells were then gently rinsed with phosphate-buffered saline (PBS) twice. The hBMMSCs were stained successively with 4,6-diamidino-2-phenylindole dihydrochloride (DAPI) (ThermoFisher Scientific, Waltham, MA, USA) and Invitrogen™ AlexaFluor™594-labeled phalloidin (ThermoFisher Scientific), at r/t in the dark for 30 min. Fluorescence images were acquired with a Nikon N-STORM confocal microscope (Nikon Corporation, Tokyo Metropolis, Japan) using NIS-Elements Viewer Software (Nikon Corporation, Tokyo Metropolis, Japan).

### 2.7. Cell osteogenic Gene Expression: RT-qPCR Assay

To determine mRNA transcript levels of the osteogenic differentiation and mineralization markers, the hBMMSCs were cultured together with the tested NPs. A real-time quantitative polymerase chain reaction (RT-qPCR) was performed to quantify gene expression. Twenty thousand HBMMSCs per well were seeded onto 12-well plates (n = 3) and incubated for 7 and 21 d with 10 µg/mL NPs-conditioned medium, in unconditioned culture medium (negative control group), or in osteogenic differentiation medium (*OsteoDiff*^®^ media; Miltenyi Biotec, Gladbach, Germany) (positive control group). Culture media and eluates were replaced every three days. The undiluted sealer-conditioned medium was prepared by immersing the previously confectioned standardized sealer discs in a culture medium (DMEM; Gibco, Gaithersburg, MD, USA) for 24 h. Total RNA from each culture was extracted using the RNeasy Mini Kit (Qiagen, Hilden, Germany) according to the manufacturer’s protocol. 1 μg of RNA was reverse transcribed for first-strand complementary DNA (cDNA) synthesis via iScript™ Reverse Transcription Supermix for RT-qPCR (Bio-Rad Laboratories Inc., Hercules, CA, USA). 

The sequences of relevant primers were as follows: (5′-3′): alkaline phosphatase or ALP (forward: TCAGAAGCTCAACACCAACG, reverse: TTGTACGTCTTGGAGAGGGC), osteonectin or ON (forward: CGGGTGAAGAAGATCCATGAG, reverse: CTGCCAGTGTACAGGGAAGATG), collagen type 1 or Col1A1 (forward: CTAAAGGCGAACCTGGTGAT, reverse: TCCAGGAGCACCAACATTAC), runt-related transcription factor 2 or RUNX2 (forward: TCCACACCATTAGGGACCATC, reverse: TGCTAATGCTTCGTGTTTCCA), bone sialoprotein or BSP (forward: TGCCTTGAGCCTGCTTCCT, reverse: CTGAGCAAAATTAAAGCAGTCTTCA), amelogenin X or AMELX (forward: CACCCTGCAGCCTCATCACC, reverse: GTGTTGGATTGGAGTCATGG), Ameloblastin or AMBN (forward: AGCCATGTTTCCAGGATTTG, reverse: TGCACCTCCTTCTTCGTTCT) [25].

Differentiation markers were measured using the expression of the housekeeping gene Glyceraldehyde 3-phosphate dehydrogenase (GAPDH) as a reference, with the following sequence (5′-3′): (forward: TCAGCAATGCCTCCTGCAC, reverse: TCTGGGTGGCAGTGATGG). To calculate the relative gene expression, the standardized 2^−ΔΔCT^ method was used [25].

### 2.8. Cell Mineralization/Calcified Nodule Formation: Alizarin Red S Staining

The mineralization or calcification ability of the hBMMSCs in contact with the tested NPs was analyzed by Alizarin Red S Staining (ARS) after 21 d of culture. The hBMMSCs were seeded onto 24-well plates at 1 × 10^4^ cells/well concentrations and allowed for attachment. The cells were then transferred into the NPs-conditioned medium and cultured for 21 d. After the culture period, the cells were fixed in 95% ethanol for 30 min at room temperature (RT), rinsed three times with double-distilled water, stained with 5% of alizarin red (pH = 4.2, Sigma Aldrich, St. Louis, MO, USA) for 5–10 min, washed repeatedly with double distilled water, and then dried at RT. The dried plate was observed under a stereomicroscope (Leica Microsystems GmbH, Wetzlar, Germany) to acquire relevant images. For quantification of the calcified nodules, the alizarin red was dissolved in 10% cetylpyridinium chloride (Sigma-Aldrich, MO, USA). After that, the plate was read at an absorbance of 405 nm by the spectrophotometric microplate reader (Thermo Fisher, USA). For this assay, both a negative control (hBMMSCs cultured in unconditioned growth medium, DMEM; Gibco, USA) and a positive control (hBMMSCs cultured in osteoinductive media (*OsteoDiff*^®^, Miltenyi Biotec, Germany) were used for reference.

### 2.9. Statistical Analysis

Statistical analyses were performed with Prism 6 (GraphPad Software, San Diego, CA, USA). Data are expressed as mean ± standard deviations (SD). The normality in the distribution of the data was previously confirmed via a Q-Q plot. Data were analyzed using one-way ANOVA and Tukey’s post hoc test. Statistical significance was set at *p* < 0.05.

## 3. Results

### 3.1. Resazurin Assay

**Figure 1:**
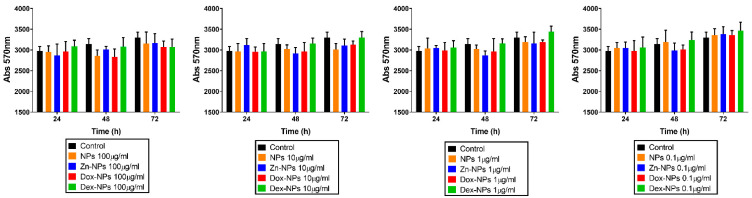
Resazurin assay results. The hBMMSCs viability was analyzed at 24, 48, and 72 h of culture in the presence of NPs. Fluorescence intensity was assessed using spectrophotometry at 570 nm. Each experiment was performed in triplicate. No differences were found among the experimental groups (*p* > 0.05).

To analyze the effects of the concentrations of the different NPs on hBMMSCs proliferation rates, a resazurin assay was carried out (Figure 1). Adequate cell viability was encountered at all NP concentrations and groups at every tested time point (24, 48 and 72 h), without significant differences if compared to the control group.

### 3.2. Cell Cycle Analysis

**Figure 2:**
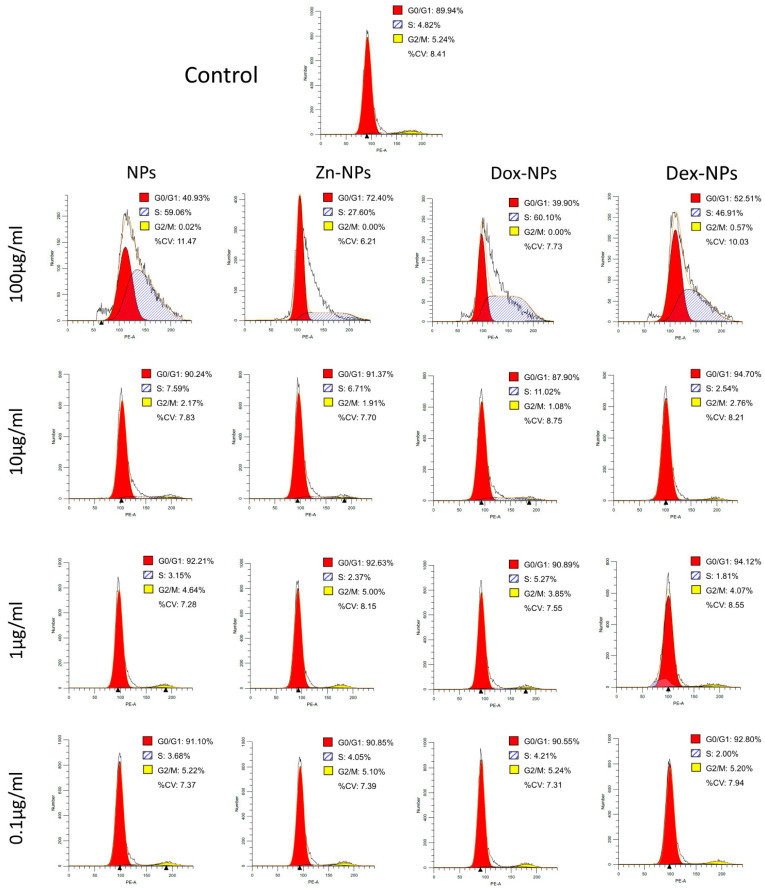
Cell cycle analysis results. The cell cycle distribution of the hBMMSCs treated with NPs detected by flow cytometry assays.

Flow-cytometry analyses of hBMMScs after 72 h of continuous treatment with different NP concentrations to monitor for potential interference with cell-cycle progression were analyzed. Cell cycle phase distributions are presented in Figure 2. At 100 µg/mL concentration, in the Dox-NPs and in the NPs groups, the majority of the cells were found in S phase (59–60%), with a moderate number of cells in G_0_/G_1_ phase (39–40%), whereas cells in the Dex-NPs group showed a similar number of cells in G_0_/G_1_ phase than in S phase (46–52%). Conversely, for NPs-treated cells, the percentage of cells in phases G_0_/G_1_, S, and G_2_/M were 72.40%, 27.60%, and 0%, respectively. At 10, 1, and 0.1 µg/mL concentrations, in all NPs groups, abundant cells in G_0_/G_1_ phase (87–92%) were encountered (Figure 2).

### 3.3. Wound Healing Assay

**Figure 3:**
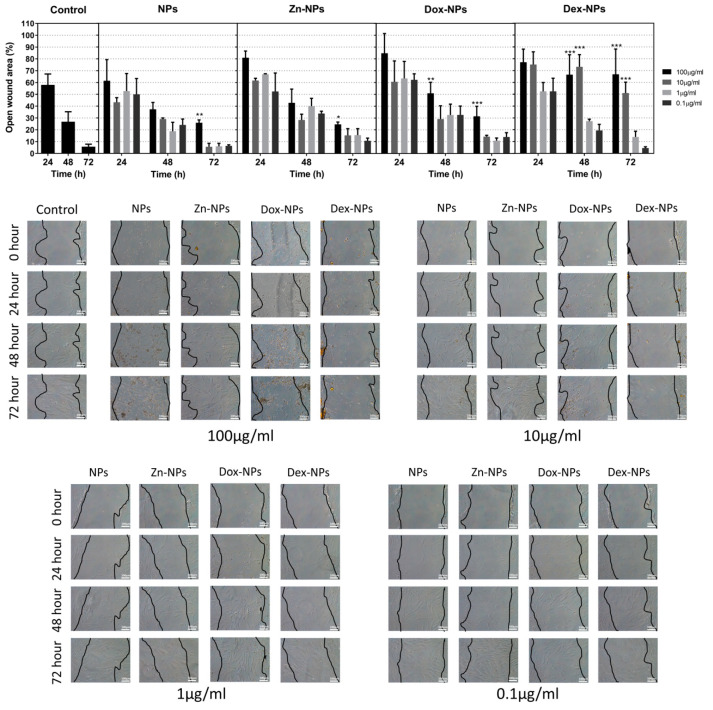
The mean and standard deviations of the different calculated wound-healing rates in the experimental groups. Representative optical microscope images of the hBMMSCs migration in the presence of the different NPs and in the control group are shown. The scale bar is 100 µm. Statistical analysis corresponds to the wound-healing rate. * *p* < 0.05. ** *p* < 0.01. *** *p* < 0.001.

Cell monolayers were wounded by a scraper and allowed to heal in the presence or absence of NPs. The cells cultured with 0.1 and 1 µg/mL concentrations showed similar behavior to that of the untreated group (control) at every time in the wound healing assay. However, the cells cultured with 100 µg/mL concentrations decreased wound closure compared to the control group after 72 h of culture (*p* < 0.001; Figure 3). Interestingly, significant differences (*p* < 0.001) were observed in the cells cultured with 10 and 100 µg/mL of Dex-NPs compared to the cells in the control group at 48 and 72 h (Figure 3).

### 3.4. Cell Cytoskeleton Labeling

**Figure 4:**
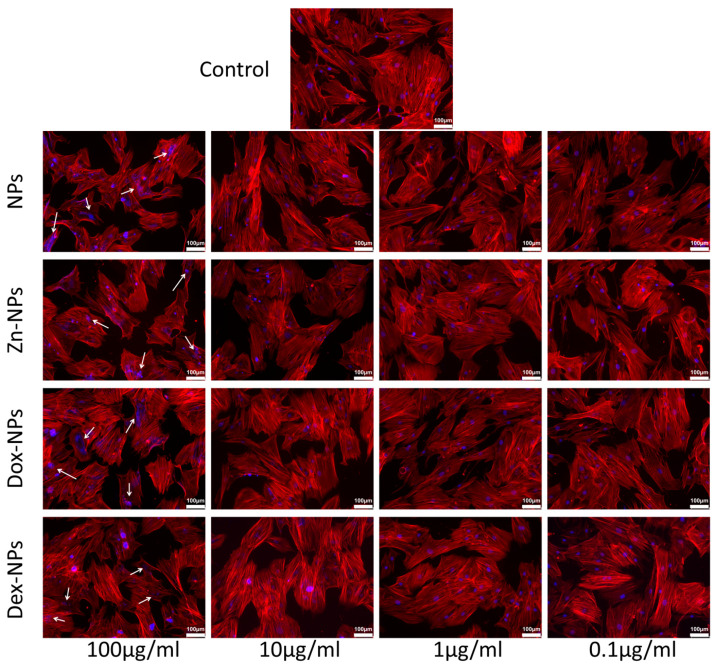
Confocal microscopy images of the cytoskeletal component distribution after AlexaFluor™ 594-labeled phalloidin and DAPI staining F-actin fibers and nucleus, respectively. Some nuclei alterations were encountered in the hBMMSCs cultured with the tested NPs at 100 µg/mL (white arrows). The scale bar is 100 μm.

Cell adhesion and morphology were investigated by staining hBMMSCs with phalloidin (red fluorescence) and DAPI (blue fluorescence) to visualize the actin cytoskeleton and cell nuclei, respectively. Immunofluorescence staining evidenced that the cells treated with the tested NPs exhibited a mesenchymal/fibroblastic cell morphology, similar to the control group. It was manifested by the regular display of F-actin, although some nuclear alterations were detected in the groups of 100 µg/mL-treated cells (Figure 4).

### 3.5. RT-qPCR Assay

**Figure 5:**
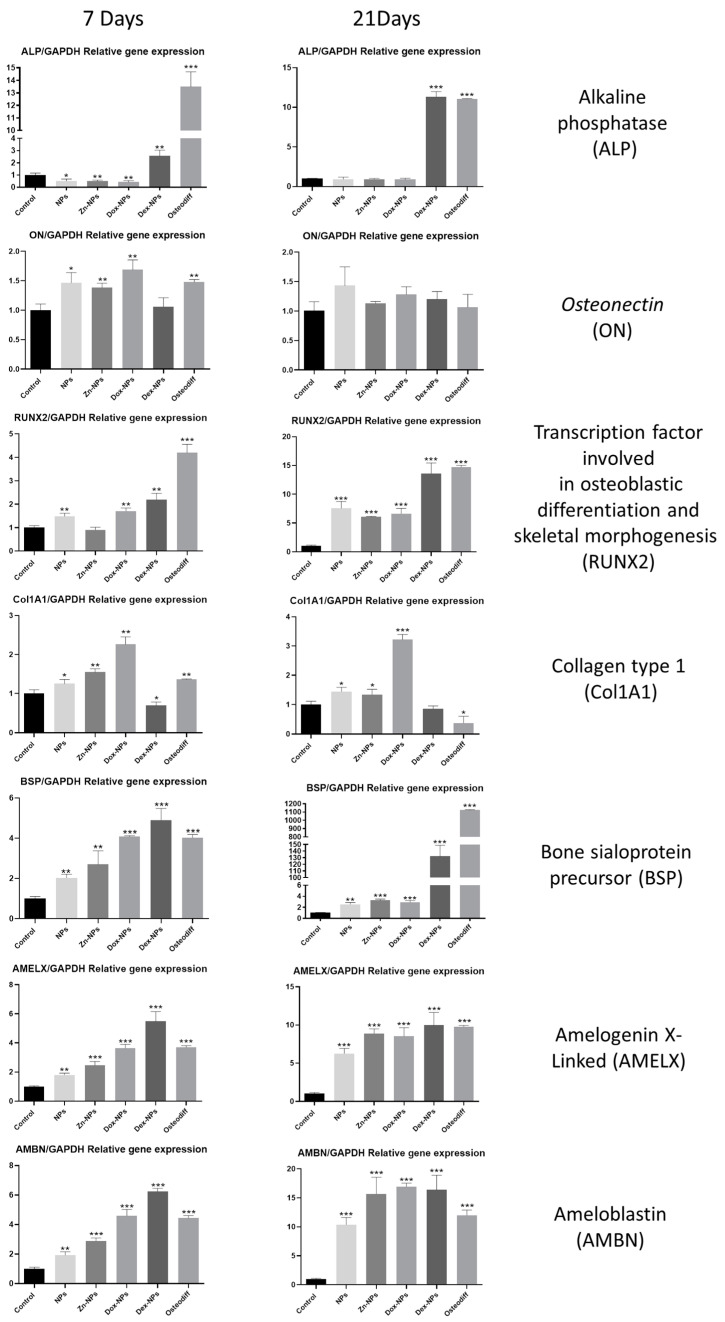
RT-qPCR assay. The mean and standard deviations of the relative mRNA expressions of the selected osteogenic-related genes (ALP, ON, RUNX2, Col1A1, BSP, AMELX and AMBN). The genes’ cell expression was determined by RT-PCR after osteogenic induction in the hBMMSCs cultured for 7 and 21 d in the presence of NPs, *OsteoDiff*^®^ media (positive control), or culture medium (negative control). * *p* < 0.05. ** *p* < 0.01. *** *p* < 0.001.

The results of the RT-qPCR assay for assessing osteogenic marker expression from the hBMMSCs cultured with the tested NPs (10 µg/mL) are presented in Figure 5.

The RT-qPCR assays evidenced a marked overexpression of ALP gen at 7, and 21 d in the presence of *OsteoDiff*^®^ media and Dex-NPs (*p* < 0.001). For ON, the cells in NPs, Zn-NPs and Dex-NPs groups presented a statistically significant increase in their expression at 7 d (*p* < 0.001) if compared to the untreated group. Regarding RUNX2, the cells in the *OsteoDiff*^®^ group attained significant overexpression (*p* < 0.001) at all time points (7 and 21 d), these values were followed by those of the cells in the Dex-NPs and in the Dox-NPs groups, which also produced significant higher expression if compared to the cells cultured in the negative control group. Interestingly, the cells in the Dox-NPs group showed a maintained overexpression of Col1A1 during all the tested time points. The cells in the Dex-NPs group attained a marked decrease in the expression of Col1A1. The cells cultured with the Dex-NPs also produced a late overexpression of BSP, AMEL, and AMBN, if compared to the negative control group.

### 3.6. Alizarin Red S Staining

**Figure 6:**
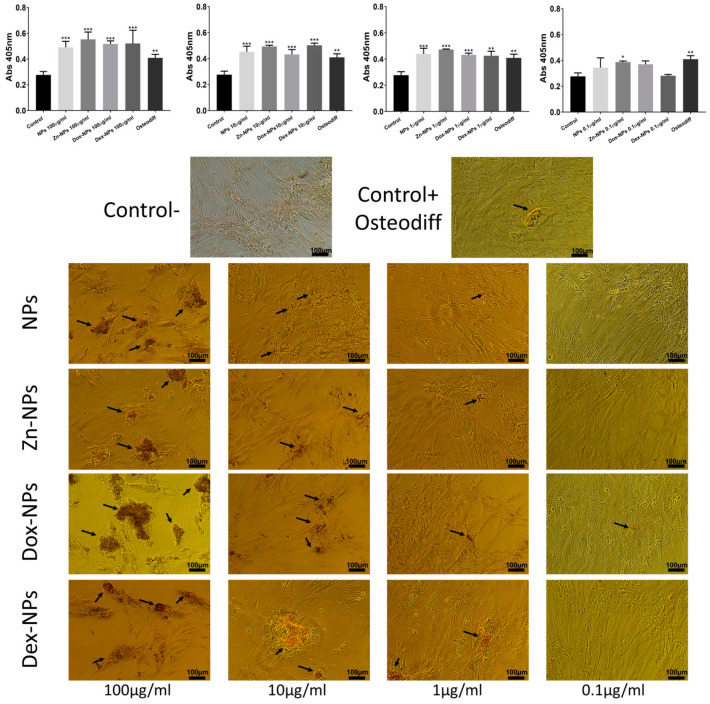
Assessment of hBMMSC calcification ability with ARS after 21 d in the presence of the experimental NPs. Some calcium deposits are visible (black arrows). The mean and standard deviations of ARS were measured by the spectrophotometric microplate reader. Representative optical images of the ARS-stained areas in the different experimental groups. * *p* < 0.05. ** *p* < 0.01. *** *p* < 0.001.

After 21 d of culturing with the tested NPs, ARS staining was performed to ascertain the cells’ calcification ability (Figure 6). The 1, 10, and 100 µg/mL-treated hBMMSCs exhibited significantly higher extensive clusters of calcium deposits than the positive and negative control groups (*p* < 0.001). The 0.1 µg/mL-treated hBMMSCs attained a similar % of ARS-stained areas if compared to the untreated group (negative control), except for those cells cultured in the Zn-NPs group (*p* < 0.05). Calcium deposits were significantly higher in the cells cultured in the *OsteoDiff*^®^ group (positive control) (*p* < 0.01) if compared to the cells grown in the untreated group (negative control).

## 4. Discussion

It has been reported that the ability of a material to enable osteogenic differentiation has crucial implications for bone regeneration [26,27]. Therefore, the present in vitro investigation aimed to evaluate the effect of the different prototypes of bioactive NPs on the viability, morphology, migration, adhesion, osteoblastic differentiation, and mineralization potential of hBMMSCs. Four different concentrations of the NPs were tested to assess the biocompatibility and bioactivity of each of the dilutions. The studied NPs should not only be able to coexist with the stem cells without negatively affecting their viability, but NPs should promote the differentiation into bone-producing cells. They should also be able to work as nano-carriers of the doped substances, thus preventing the biological washout that would happen if these substances were applied as free drugs. The tested particles have a mean hydrodynamic size of approximately 250 nm; therefore, the employed term of nanoparticles may be questioned as they exceed the size named in the conventional definition of nanoparticles (1–100 nm). However, under the current regulations, it is considered that the nanomaterials may be larger than 100 nm if they possess size-dependent properties because they are not readily predictable based on a simple size scaling [28].

hBMMSCs were selected for the study as they are precursors of osteoblastic lineages. This in vitro study design offers a consistent analysis of the main biological properties of the NPs cultured together with cell populations that would be in contact with the biomaterials during their clinical use. This may preliminarily predict their clinical behaviour [29]. Different tests were used in order to achieve the proposed objectives.

Resazurin was employed to ascertain the in vitro biocompatibility of the cells in the short term. This test revealed stable biocompatibility up to 72 h for the four prototypes of NPs regardless of the NP concentrations (Figure 1). Some previous studies have reported a dose-dependent cytotoxic effect of doxycycline exerted on bone marrow stromal cells after 24–48 h of culturing [30,31]. Although the toxic dose of doxycycline has not been definitively established, it is clear that the sustained release of doxycycline of the present NPs remains inside the non-cytotoxic dosage of this antibiotic. The same conclusion could be drawn with the other two doping substances. It should be considered that these NPs are composed of 2-hydroxyethyl methacrylate, ethylene glycol dimethacrylate and methacrylic acid, connected covalently. 

Altogether, the synthesis is characterized by a simple but efficient procedure in which the absence of toxic solvents or non-polymerized compounds is crucial, which are likely to later interfere with cellular biological processes [15]. Furthermore, the last step in NPs fabrication is the removal of residuals by two consecutive washing procedures in methanol. Previous studies suggested that material size and surface area play important roles in the observed cytotoxicity, and these effects are generated in the cells by NPs, but not by the ions or molecules included in the medium. The NPs may then result in more cytotoxicity compared with the larger ones (size effect) [32]. It is important to stress the absence of cytotoxic effects of the tested NPs.

With the cell cycle analysis (Figure 2), the objective was to observe if the NPs or any of the doped compounds affected the cellular cycle of the stem cells. In order to analyze the cell cycle phase distributions, the profiles of the treated cells were compared to a control without NPs. After 72 h of culturing, the cells treated with 100 µg/mL NPs suffered a noticeable alteration in the cellular cycle. As can be seen in Figure 2, in all the NP types, the cellular cycle appears to be affected; thus, this toxic event could be attributed to the dose of the NPs rather than to one of the active substances doped onto them. The hBMMSCs treated with this concentration were somehow not able to divide (nearly 0% of the cells were in G2/M phases), leading to an arrest of cells in the S phase (ranging between 27–60%). These percentages were far from those attained by the control group: 5.24% and 4.82%, respectively. The increase of cells in these two phases was at the expense of reducing the percentage of cells in the G_0_/G_1_ phase. 

The rest of the concentrations of NPs did not apparently affect the cell cycle profile, regardless of the doped substance. The results are verified by the ones obtained when analyzing the cytoskeleton structure by phalloidin staining (Figure 4). With this technique, we have been able to check the structural integrity of the treated cells. Nevertheless, when observing the fluorescent images of the groups where the NPs were used at 100 µg/mL, nuclear abnormalities became evident. These discrepancies could be associated with the accumulation of cells in the S phase, wherein the DNA is synthesized. Since the cells continued to produce genetic material but could not divide, these genetic substances accumulated inside the cells’ nuclei, leading to the visualization of nuclear anomalies by staining of the F-actin. In addition to this punctual anomaly, when treated with 100 µg/mL NPs, and following the trend previously described, there are no apparent deviations from normality regarding the nuclear and cellular morphology of the rest of the experimental groups. The cells mainly display an elongated and spread morphology, with evident similarities to the cells cultured in the control group (Figure 4). If the cell’s viability, cytoskeleton, and proliferation analysis are considered all together, the data revealed no identification of necrotic and/or apoptotic effects of the different NPs. The exceptions were Dex-NPs and the Dox-NPs, which were found to present a potentially dose-dependent but low cytotoxic effect (Figure 4).

Some bioactive materials may release components and substances that could perhaps delay or enhance the healing potential of the affected tissue [33]. This is the reason why the wound healing assay was performed. Therefore, how the hBMMSC would react after the injury of their 3D matrix in the presence of NPs was analyzed. At 10 µg/mL and 1 µg/mL concentrations, no statistical differences were found between the migration of the cells grown in the control group and the ones treated with the different types of NPs, except for the group of Dex-NPs, in which at the concentration of 10 µg/mL, after 48 and 72 h of growth, the migration was significantly reduced (Figure 3). This fact could be due to the anti-inflammatory effect of this glucocorticoid, since the repair processes of the tissues are achieved through a balance between inflammatory and anti-inflammatory mediators. This effect of the treatment with Dex-NPs may be solved when reducing the dosage to 1 or even to 0.1 µg/mL. However, it should be noted that many other factors may influence in vivo tissue repair processes. Among the variety of cells, macrophages play a pivotal role in bone healing and regenerative processes. There is a close cross-talk between macrophages and hBMMSCs [34]. Previous in vitro studies have demonstrated that M1 macrophages, by secreting oncostatin M, were capable of promoting osteoblastogenesis of the MSCs [34,35] and, thus, bone repair. Conversely, other studies reported on the M2 phenotype, which enhances osteogenic differentiation of bone marrow stem cells [34,36]. In this regard, the application of dexamethasone, given its potent immunomodulatory capacity, would play an important role in the cross-talk between both types of cells. This critical point deserves future research.

As can be observed in Figure 3, the hBMMSCs treated with Zn-NPs exerted virtually the same migration rates as the control. This is not the first time in which Zn has been employed as a stimulator of MSCs differentiation and osteogenic promoter. It has also been suggested that the action of zinc on osteogenesis would be, in part, due to its immunomodulation capacity. Although it has been demonstrated that zinc can regulate the osteoimmune microenvironment in order to promote osteogenesis, the convenient concentration range still needs to be further determined [37]. Bai et al. [38] reported a dual action of Zn: (i) first, in the early phases of healing (1 to 3 d), Zn triggered macrophage polarization into inflammatory phenotype; (ii) subsequently, after 3 d, this phenotype shifted into a M2 subtype, leading to the anti-inflammatory environment. Apart from the previously mentioned actions of zinc, the osteogenic potential of this ion could also be influenced by its ability to suppress the formation of osteoclasts by inhibiting the osteoblasts production of receptor activator of nuclear factor kappa-Β ligand (RANKL) [37,39]. The present Zn-NPs have also shown a potent antibacterial effect against an in vitro periodontal biofilm when applied on hydroxyapatite [17] and titanium surfaces [8], potentially contributing to a relatively aseptic area and thus leading to an anti-inflammatory environment. 

Osteoblastic differentiation of the hBMMSCs has also been assessed by quantification of the expression of the main differentiation-related genes by means of RT-qPCR (Figure 5). Runx-2 is a member of the runt homology domain transcription factor family, and plays an important role in osteoblast differentiation [40,41] and, together with ALP, are the most frequently used markers of early osteoblasts differentiation. After seven days, both genes were upregulated by all types of NPs if compared with the control. Conversely, only Runx-2 maintains this up-regulation after 21 days for all the study groups. At this point, the expression of both Runx-2 and ALP of the Dex-NPs treated cells attained statistically identical values to those obtained by the cells cultured in *Osteodiff*^®^ media, an optimized differentiation medium to generate osteoblasts from human MSCs. These results are in accordance with previous investigations, in which rat bone marrow-derived mesenchymal stem cells were cultured in the presence of dexamethasone and the expression of Runx-2, ALP, and OPN were significantly increased [42]. Other authors have also proven, in vitro, the capacity of dexamethasone to increase the expression of other differentiation-related genes of osteoblastic lineages [43,44].

Collagen type I (Col-I) and bone sialoprotein (BSP) are some of the most representative components of extracellular matrix found in bone and dentin. Col-I is the main component of the organic part, and BSP has been suggested to act as a mineralization nucleus for the deposition of the first apatite crystals during the mineralization process [45]. Both of these genes were overexpressed after being exposed during 7 d to all the types of NPs (Figure 5). After 21 d, the cells cultured in the presence of Dex-NPs, attained a greater expression of BSP than the rest of the study groups. This result is in accordance with the one reported by Nguyen et al. [46], who cultured hMSCs over poly (L-lactic acid) nanofibers with and without dexamethasone, and found that the BSP expression was greatly increased in the presence of the corticoid.

Amelogenins and ameloblastins are the main representatives of the enamel matrix proteins. Clinically, enamel matrix proteins from a porcine origin are being used since they have demonstrated the ability to advance and enhance regeneration of the periodontal tissues [47], since it is thought that they are able to initiate events that occur during the growth of the periodontal tissues [48]. Thus, the expression of the genes encoding for these proteins was evaluated in the present in vitro model. It is noteworthy that both amelogenin X-Linked (AMELX) and amelobastin (AMBN) show a parallel expression among the different study groups (Figure 5). The expression of these two genes when the cells were exposed to the NPs was, in all cases, similar to or even higher than the results attained by the cells cultured in the osteogenic *OsteoDiff*^®^ media. So, this effect could be attributed to the intrinsic structure and components of the NPs. However, after 21 d the highest values were obtained by the cells of the Dex-NPs and the Dox-NPs groups (Figure 5).

All the NP concentrations (except for 0.1 µg/mL) attained Alizarin staining results similar to *OsteoDiff*^®^ (Figure 6). It may be explained as these NPs have been shown to be bioactive (able to accumulate calcium and phosphate complexes on their surfaces if immersed in simulated body fluid solutions) [16]. It is produced because NPs exhibit carboxyl groups on their surface, which easily chelates calcium [16]. This biomimetic remineralization may be crucial in alveolar bone regeneration, as calcium phosphate deposits are able to stimulate cells, leading to the formation of bone [49]. During bone metabolism, osteoclasts release Ca^2+^ and PO_4_^2−^ derived from the mineralized matrix, causing a local increase in ion concentrations in the microenvironment, which plays a role in osteoblast proliferation and differentiation. Increases in extracellular Ca^2+^ concentrations are potent chemical signals for osteoblasts cell migration and growth [49], and for bone remodeling [50]. There are biological regulatory mechanisms underlying the intracellular and extracellular calcium concentrations [49].

As a study limitation, it should be taken into account that one single cell line was used, and it may not necessarily be extrapolated to other cell types; however, these NPs have been previously shown to be non-cytotoxic to human fibroblasts [16]. Moreover, a different nanostructured material (woven nanofibres) with the same polymeric composition and loaded with doxycycline were not only found to be non-toxic but also osteogenic when cultured with an osteoblasts cell line [41].

To the best of our knowledge, it is the first time that such a complete in vitro cell study has been performed, demonstrating the increase of the differentiation potential of stem cells in the presence of doxycycline or dexamethasone-doped NPs. Therefore, a step forward in the research about the potential use of these Dex-NPs and Dox-NPs in periodontal and alveolar bone regeneration is encouraged. Taking into account the complex and multifactorial processes occurring in these diseases, an animal model should be designed for further testing.

## 5. Conclusions

Despite the limitations of the present study, Dex-NPs and Dox-NPs exhibited cytocompatibility, osteogenic potential and wound healing ability promoting hBMMSC differentiation into osteogenic lineages. Future investigations, both in vitro and in vivo, are required to confirm the suitability of these NPs for their clinical application.

## Data Availability

The data presented in this study are available on request from the corresponding author.
